# What is a Rhythm for the Brain? The Impact of Contextual Temporal Variability on Auditory Perception

**DOI:** 10.5334/joc.344

**Published:** 2024-01-17

**Authors:** Pierre Bonnet, Mathilde Bonnefond, Anne Kösem

**Affiliations:** 1 Lyon Neuroscience Research Center (CRNL), Computation, Cognition and Neurophysiology team (Cophy), Inserm U1028, Université Claude Bernard Lyon1, CNRS UMR 5292, 69000 Lyon, France

**Keywords:** Temporal predictions, auditory perception, probabilistic temporal contexts, temporal statistics, rhythmicity perception

## Abstract

Temporal predictions can be formed and impact perception when sensory timing is fully predictable: for instance, the discrimination of a target sound is enhanced if it is presented on the beat of an isochronous rhythm. However, natural sensory stimuli, like speech or music, are not entirely predictable, but still possess statistical temporal regularities. We investigated whether temporal expectations can be formed in non-fully predictable contexts, and how the temporal variability of sensory contexts affects auditory perception. Specifically, we asked how “rhythmic” an auditory stimulation needs to be in order to observe temporal predictions effects on auditory discrimination performances. In this behavioral auditory oddball experiment, participants listened to auditory sound sequences where the temporal interval between each sound was drawn from gaussian distributions with distinct standard deviations. Participants were asked to discriminate sounds with a deviant pitch in the sequences. Auditory discrimination performances, as measured with deviant sound discrimination accuracy and response times, progressively declined as the temporal variability of the sound sequence increased. Moreover, both global and local temporal statistics impacted auditory perception, suggesting that temporal statistics are promptly integrated to optimize perception. Altogether, these results suggests that temporal predictions can be set up quickly based on the temporal statistics of past sensory events and are robust to a certain amount of temporal variability. Therefore, temporal predictions can be built on sensory stimulations that are not purely periodic nor temporally deterministic.

## Introduction

Temporal predictions are believed to play a key role in the way we process sensory information (Jones, 1976; Schroeder & Lakatos, 2009; Arnal & Giraud, 2012; Nobre et al., 2012). Predicting the timing of future sensory events allows to allocate cognitive resources at the expected time of occurrence, and therefore facilitates the sensory processing of these upcoming stimuli (Jones & Boltz, 1989; Large & Jones, 1999; Nobre et al., 1999). As a consequence, the perception of sensory events is improved when their timing is fully predictable. Auditory discrimination performances are also improved when the temporal context of the stimulation is deterministic, as when auditory stimulation is periodic (Cravo et al., 2013; Jaramillo & Zador, 2010; Lawrance et al., 2014; Morillon et al., 2016; Rimmele et al., 2011), when temporal intervals are repeated (Breska & Deouell, 2017), or when the temporal intervals are slowing decreasing or increasing at a predictable pace (Cope et al., 2012; Morillon et al., 2016).

However, from a naturalistic point of view, temporal contexts are rarely fully isochronous nor deterministic. Speech acoustic signals in particular presents complex statistical temporal regularities (Singh et al., 2003; Cummins, 2012; Varnet et al., 2017) that are supposedly used to form temporal expectations and influence language comprehension (Tillmann, 2012; Jadoul et al., 2016; Kösem & Van Wassenhove, 2017; Kösem et al., 2018; Aubanel & Schwartz, 2020). How temporal predictions occur in non-fully predictable temporal contexts such as speech and music and how they influence auditory perception is still under debate (Jadoul et al., 2016; Herbst & Obleser, 2017, Zoefel & Kösem, 2022). During speech listening in particular, temporal prediction mechanisms are put forward as an important mechanism that would contribute to acoustic segmentation and enhanced processing of relevant auditory information (Giraud & Poeppel, 2012; Meyer et al., 2019; Peelle & Davis, 2012; Zoefel & Kösem, 2022). In line with this, temporal predictability based on rhythmic cues present in the signal, specifically on the average speech rate, influences speech perception (Dilley & Pitt, 2010; Kösem et al., 2018). Yet, speech processing also requires to take into account probabilistic temporal variations in syllable and word durations naturally present in languages (Jadoul et al., 2016; Varnet et al., 2017; Ten Oever & Martin, 2021). Probabilistic inference of sensory timing influences explicit temporal judgments of auditory events (Cannon, 2021; Doelling, 2021), tapping (Cannon, 2021), warned reaction time tasks (Los et al., 2017), responses times during auditory discriminations tasks (Herbst & Obleser, 2017). However, it is unclear to what extent probabilistic temporal predictions influence the discrimination of sounds per se. The aim of this study is therefore to investigate how the temporal statistics of auditory stimulation influences ongoing auditory perception, specifically when the temporal context is not fully predictable in time. Additionally, the perception of rhythmicity, here defined as the perception of how temporally regular the sound sequences are, is known to vary across participants (Geiser et al., 2009; Krause et al., 2010; Repp, 2010; Fiveash et al., 2022). Subjective perceived rhythmicity may have an influence on the way temporal predictions are formed (Doelling & Poeppel, 2015): if participants rely on an internal model of temporal predictions that differs from external timing, maximal auditory discrimination performance would occur when participants judge the temporal context to by maximally predictable, and not necessarily when the external context is regular. We therefore also explored whether auditory perception is influenced by the subjective perception of the contextual temporal structure.

**Figure 1:**
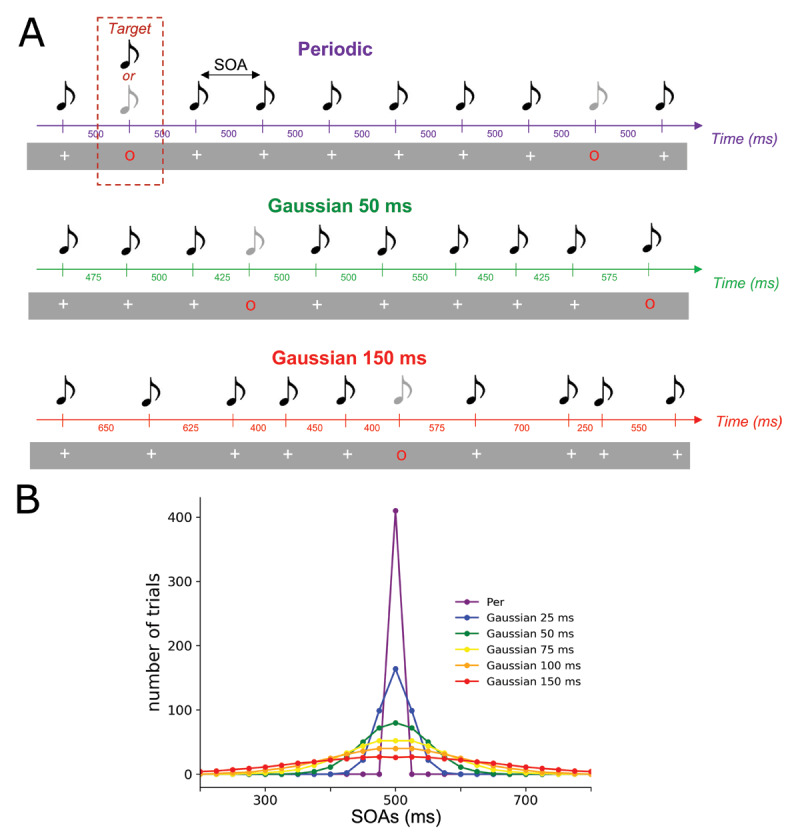
**Experimental design. (A) Example of three sequences of different temporal STD used in this experiment**. Each sequence consisted of a stream of simultaneous auditory and visual stimuli. A standard stimulus corresponded to a 440 Hz pure tone co-occurring with a white cross. Occasionally a red circle appears in the stream indicating a target stimulus, on which participants had to discriminate between a standard (440 Hz) and deviant (220 Hz) pure tone. **(B) Distribution of SOAs in each sequence.** For each sequence, the distributions of the SOAs were drawn of Gaussian distributions with equals means (500 ms) but distinct STDs. Six conditions were designed: from 0 (periodic) to 150 ms of STD with data points built from 100 ms to 900 ms and spaced from 25 ms.

To do this, we used an auditory oddball paradigm adapted from the study of Morillon and colleagues (2016). Participants were asked to detect deviant sounds that where embedded in 3 min-long sound sequences. The Stimulus-Onset-Asynchrony (SOA) between each sound of the sequence was drawn from Gaussian distributions. The distributions had the same mean (500 ms) but different standard deviations (STD): from 0 ms (periodic) to 150 ms STD (Figure 1B). Results suggest that (i) temporal predictions can be formed in aperiodic probabilistic context, though auditory discrimination performance progressively declines with the temporal variability of a context, (ii) these temporal prediction effects are set up quickly from the local temporal statistics of the context. Therefore, this work suggests that temporal prediction mechanisms are robust to temporal variability, and that temporal predictions built on sensory stimulations that are not purely periodic nor temporally deterministic can influence auditory perception.

## Materials and Methods

### Participants

Twenty-three participants (11 females, mean age = 25.6 years, 3 left-handed) took part in the experiment. Participants reported no history of neurological or psychiatric disease, normal hearing and normal or corrected-to-normal vision. Four participants had outlier data and were excluded from data analysis: one participant responded at chance level throughout the experiment, three participants had outlier subjective rhythmicity ratings of the sequences (±1.5 interquartile range of regression scores) (Figure S1). Therefore, nineteen participants were included for the analysis. The study was approved by an ethical committee (CPP) and all participants signed a written consent and received payment for their participation.

### Stimuli

Participants heard sequences of pure tones, that were either a standard sound (corresponding to a pure 440 Hz sound) or a deviant sound (pure 220 Hz sound). The sounds were presented via headphones for 100 ms (with 5 ms ramp-up and ramp-down in volume). With each sound, visual cues were presented via a digital display (1600, 1024 resolution; 120 Hz refresh rate) and were displayed in front of them (70 cm) in the center of the screen for a duration of 100 ms. Visual cues were synchronized to appear simultaneously with the sounds. A “red circle” visual cue indicated a target and that the participant had to respond to this trial by pressing a button (Figure 1A). A “white cross” cue indicated a standard trial of the context and that participants did not have to respond to this trial. When the “white cross” was presented on the screen, the synchronized sound was always a standard sound whereas when the “red circle” was presented, the synchronized sound could either be a standard or a deviant sound (with equal 50% probability). The “red circle” stimulus was used to indicate to the participant that the sound was a target stimulus and that an answer was required. With this manipulation, we could make sure that the response of the participant was referring to the last cued sound, and not to any other sound from the sequence (which is presented continuously at a relatively fast rate around 2 Hz). In the absence of the visual cue, we could not unambiguously decipher to which sound the participant was reacting to. In addition to the pure tones, broadband white noise was presented continuously to make the task more difficult. The signal-to-noise ratio between pure tones and white noise was adjusted individually via a staircase procedure (see Procedure). All stimuli were generated and presented via the Psychophysics-3 toolbox.

### Procedure

The experiment was composed of 12 blocks of 3 min 30 s. Each block consisted of a sequence of auditory sensory stimuli masked in constant noise. Participants had to discriminate the sound when a red visual cue was presented on the screen (target trial) (Figure 1A). To vary the temporal regularities of the context, the SOAs between the sounds of each block was drawn from distinct distributions. In the Periodic condition, the SOA was fixed at 500 ms. For the Gaussian conditions, the SOAs were drawn from Gaussian distributions with distinct STDs of 25 ms, 50 ms, 75 ms, 100 ms and 150 ms (Figure 1B). The SOAs data points used to build these gaussian distributions were spaced out every 25 ms to allow accurate sampling of these conditions. Both standards and target trials were drawn from these distributions. Each block consisted of 410 trials including 56 target trials. Between two target trials, a minimum of 4 standard sounds and a maximum of 10 standard sounds (uniform distribution) could occur. The target trials could not appear in the first 10 trials of the sequence. Two blocks were presented for each condition and the block order was pseudo-randomized so that the same condition was not presented twice in succession. Therefore, 112 target trials were obtained for each condition.

After each block, the subjective perception of the rhythmicity of the temporal context was assessed: participants were asked to rate the global rhythmicity of the sequence. We specifically asked to rate whether the sounds in the sequence were presented at a regular pace on a scale from 0 (totally not rhythmic) to 10 (totally periodic). Before the main experiment, a staircase procedure was performed to adjust the signal-to-noise ratio (SNR) so that the average sound discrimination performance was within ~80% correct responses (mean SNR = –14.9 dB, within [–16.0, –13.7] dB range). These SNRs are slightly higher than previously reported detection thresholds of pure tones (around –17 dB) (McPherson et al., 2022). We think for this reason that participants were able to hear both standards and deviant sounds and that our task relied on pitch discrimination. In the staircase, 75 sounds were displayed with periodic SOAs (500 ms) and targets trials could appear every ~2–3 tones.

### Data analysis

Generalized linear mixed models (GLMMs) were computed using lme4 (version 1.1-28) (Bates et al., 2014) with R 4.1.2 (2021-11-01), on both the subject’s responses (1 for a correct response, 0 if incorrect, binomial distribution) and subject’s response times (gamma distribution) as dependent variables. We first included the global temporal STD (global STD, continuous variable) as fixed effect, and the factor Subject as a random effect. Stepwise models comparison was done using the likelihood ratio test, and Type II Wald chi-square tests were used to assess the best model fit, and the significance of fixed effects (Bates et al., 2014; Luke, 2017). For subjects’ responses, the best model only included the Subject random intercept (as adding the random slope did not significantly improve the model’s explained variance: Chi-square = 2.72, p = 0.2557). For the response times, the best model included both random intercepts and random slopes. To evaluate the difference in performance between each Global STD condition in our experiment, we then performed post-hoc tests using the emmeans package version 1.7.4.1. For this we considered Global STD as a categorical factor and compared each Global STD level (0, 25, 50, 75, 100, 150 ms) using Tukey multiple comparison correction. As exploratory analyses, we investigated the impact of the subjective perception of rhythmicity on performances. To do this, we compared the first models to a new model that included the predictor Rhythmicity Rating (gaussian distribution) as additional fixed effect. We also investigated the correlation between Global STD and Rhythmicity Rating. For this, Rhythmicity Rating was considered as a dependent variable and Global STD as fixed effect, and Subject as Random effect.

We also investigated how the recent temporal statistics in the non-periodic sequences impacted performance (i.e. based on the statistical distribution of the SOAs between the last N sounds before target presentation). To do this, we computed for each participant 2-D plots representing the discrimination accuracy and response times according to the STD and mean of the N previous SOAs. Specifically, we computed the mean and STD of the local SOA distribution drawn from the N previous SOAs before each target trial (with N ranging from 1 to 7 SOAs for the mean SOA, and N ranging from 2 to 7 SOAs before target trial for the SOA STD). We then binned target trials per SOA distribution mean (from 400 to 600 ms SOA mean, with a sliding window of ±20 ms length) and per SOA distribution STD (from 10 ms to 100 SOA STD, with a sliding window of ±10 ms length). Bins containing less than 5 trials per participant were excluded from further analysis. For each bin, we computed the average accuracy and response time across trials. We obtained a 2-D plots representing how the mean and STD of the 2/3/4/… last SOAs impacted accuracy and response times for each participant. We investigated whether, across participants, performances would be relatively better or worse depending on the mean or STD of the local temporal statistics. To test this, we therefore Z-scored the 2-D plots for each participant and applied cluster-based permutation statistics (using MNE version 1.0.3) to the z-scored data (Maris & Oostenveld, 2007). One sample t-tests against zero were computed for each sample. Adjacent samples with a p-value associated to the t-test of 5% or lower were selected as cluster candidates. The sum of the t-values within a cluster was used as the cluster-level statistic. The reference distribution for cluster-level statistics was computed by performing 1000 random sign-flipping permutations of the data. Clusters were considered significant if the probability of observing a cluster test statistic was below the 2.5-th quantile and above the 97.5-th quantiles for the reference distribution. The choice of the cluster permutation tests was done to solve multiple comparison testing, and was motivated by the fact that we had two-dimensional data whose adjacent samples were correlated in both dimensions (i.e. we expected that samples with close mean SOA and close SOA STD would lead to similar performances; similarly, we expected that the computation of the mean and STD of the N preceding SOAs would also correlate with the mean and STD of the N-1, N-2 SOAs, and so forth). Additionally, cluster-based non-parametric permutation testing allows to capture nonlinear effects (expected to occur for mean SOA effects in particular). Finally, to further evaluate the relative contribution of global and local temporal STD on performances, we ran GLMMs that included both Global STD and the Local STD (from the N previous SOAs, N rating between 2 to 7), and the last SOA before target presentation as predictors of subject’s response and response times (Table S1).

## Results

### Temporal variability impacts auditory discrimination accuracy and response times

**Figure 2:**
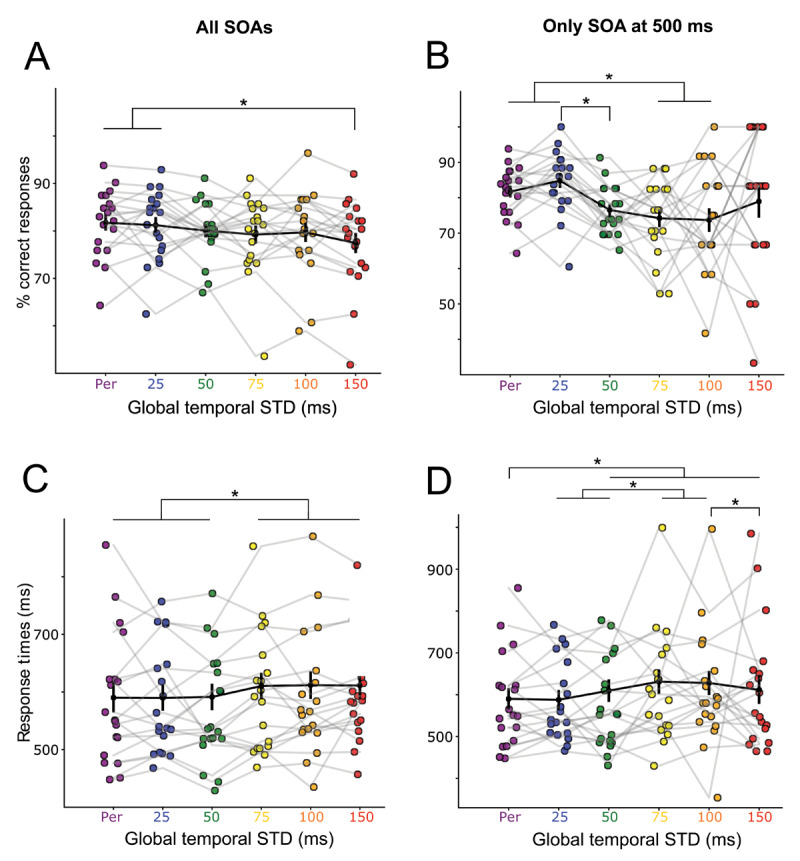
**Auditory deviant discrimination is influenced by the temporal variability of the sound sequences. (A)** Percentage of correct responses and **(C)** Response times as function of the standard deviation of SOAs in the auditory sequences. Each color dot represents a participant. Black dots represent the average across participants. Error bars indicate the Standard Error of the Mean (SEM) and stars indicate significant differences (*p* < 0.05). **(B)** Percentage of correct responses and **(D)** Response times restricted to target trials presented at SOA = 500 ms (mean of the distributions).

We tested the effect of the temporal variability of auditory sequences on auditory accuracy and on response times. Participants auditory discrimination significantly decreased as a function of contextual temporal variability (main effect of the factor Global STD (*χ2*(1) = 14.574, *p* = 0.0001)). Discrimination accuracy was highest in the periodic context, and progressively decreased with increasing global temporal STD (accuracy decreased by 0.6% every 25 ms). Contrasting each global STD condition between one another, post-hoc tests revealed that accuracy in the periodic condition was statistically different from the more aperiodic condition (difference % correct responses Periodic – Gaussian 150 = 4.28%, *p* = 0.0061). Moreover, performance was also statistically different between the contexts Gaussian 25 and Gaussian 150 (difference % correct responses Gaussian 25 – Gaussian 150 = 3.76%, *p* = 0.0262).). These results suggest that the percentage of correct responses is higher in conditions with less variable contexts even if they are not completely periodic (e.g., in the Gaussian 25 condition) (Figure 2A).

Response times were also significantly affected by the temporal STD of the context (main effect of the factor Global STD (*χ2*(1) = 115.47, *p* < 0.0001)). Response times were faster in temporal contexts with low variability and progressively slowed as the global temporal STD increased (response times increased by 5 ms as the context variability increased by 25 ms). Post-hoc tests showed that the three conditions with the lowest temporal variability: Periodic, Gaussian 25 and Gaussian 50 were statistically different from the three conditions with the highest temporal variability: Gaussian 75, Gaussian 100 and Gaussian 150 (difference response times (Periodic – Gaussian 75 = –20.2 ms, *p <* 0.0001); (Periodic – Gaussian 100 = –21.6 ms, *p <* 0.0001); (Periodic – Gaussian 150 = –21.2 ms, *p <* 0.0001); (Gaussian 25 – Gaussian 75 = –20.8 ms, *p <* 0.0001); (Gaussian 25 – Gaussian 100 = –22.1 ms, *p <* 0.0001); (Gaussian 25 – Gaussian 150 = – 21.8 ms, *p <* 0.0001); (Gaussian 50 – Gaussian 75 = –19.3 ms, *p =*0.0001); (Gaussian 50 – Gaussian 100 = –20.7 ms, *p <* 0.0001); (Gaussian 50 – Gaussian 150 = –20.3 ms, *p =*0.0003). Results on response times suggest that there is a gap between contexts with low temporal variability and contexts with global temporal STD that exceed 75 ms (Figure 2C).

The more variable the auditory sequence, the more variable the target stimuli’s SOA. It could therefore be possible that the preceding results only reflect the impact of target’s SOA variability, and not of the overall temporal context. In particular, perception is subject to temporal hazard rate, so that auditory discrimination performances improve the longer you wait for the stimulus (Herbst & Obleser, 2019), and reversely, auditory perception performance could decrease drastically for shorter SOAs. To alleviate these effects, we restricted our analyses to all targets whose preceding SOA were of 500 ms only. We still observed similar effects of temporal context as when all SOAs were included. Participants auditory discrimination significantly decreased as a function of contextual temporal variability (main effect of the factor Global STD (*χ2*(1) = 14.490, *p* = 0.0001409)). Contrasting each global STD condition between one another, post-hoc tests revealed that accuracy in the periodic condition was statistically different from the condition Gaussian 75 ms and from the condition Gaussian 100 ms (difference % correct responses Periodic – Gaussian 75 = 7.50%, *p* = 0.0174; Periodic – Gaussian 100 = 8.04%, *p* = 0.0371). Condition Gaussian 25 ms was also significantly different from the conditions Gaussian 50 ms, Gaussian 75 ms, and Gaussian 100 ms (difference % correct responses Gaussian 25 – Gaussian 50 = 8.27%; *p* = 0.0036; Gaussian 25 – Gaussian 75 = 10.59%; *p* = 0.0004; Gaussian 25 – Gaussian 100 = 11.13%; *p* = 0.0013). These results suggest that when we took only the SOA at the mean of the distributions (500 ms) auditory accuracy was also better in low variability conditions (e.g., periodic and Gaussian 25 ms) compared to conditions with more variability in the context (e.g., Gaussian 75 and 100 ms) (Figure 2B).

For the response times, there was also a significant main effect of the factor Global STD (*χ2*(1) = 77.926; *p* < 0.0001). Response times were also faster in temporal contexts with low variability and progressively slowed as the global STD increased. Post-hoc tests show that the Periodic condition was different from the Gaussian distributions above 50 ms STD (Periodic – Gaussian 50 = –19.6 ms, *p* < 0.0218; Periodic – Gaussian 75 = –41.1 ms, *p* < 0.0001; Periodic – Gaussian 100 = –38 ms, *p* < 0.0001; Periodic – Gaussian 150 = –21.5 ms, *p* < 0.0218). RTs in Gaussian 25 ms and Gaussian 50 ms were also significantly different from the Gaussian 75 ms and Gaussian 100 ms conditions (Gaussian 25 – Gaussian 75 = –43.3 ms, *p* < 0.0001; Gaussian 25 – Gaussian 100 = –40.2 ms, *p* < 0.0001; Gaussian 50 – Gaussian 75 = –21.4 ms, *p* < 0.0432; Gaussian 50 – Gaussian 100 = –18.3 ms, *p* < 0.0001). RTs in the conditions Gaussian 100 and Gaussian 150 were also statistically different (Gaussian 100 – Gaussian 150 = –16.4 ms, *p* < 0.0288). Results on response times with the SOA at 500 ms only also shows differences between low variability contexts (e.g., Periodic, Gaussian 25 ms or 50 ms) and contexts with more variability in the global temporal STD (Figure 2D).

### Statistical temporal predictions occur rapidly

**Figure 3:**
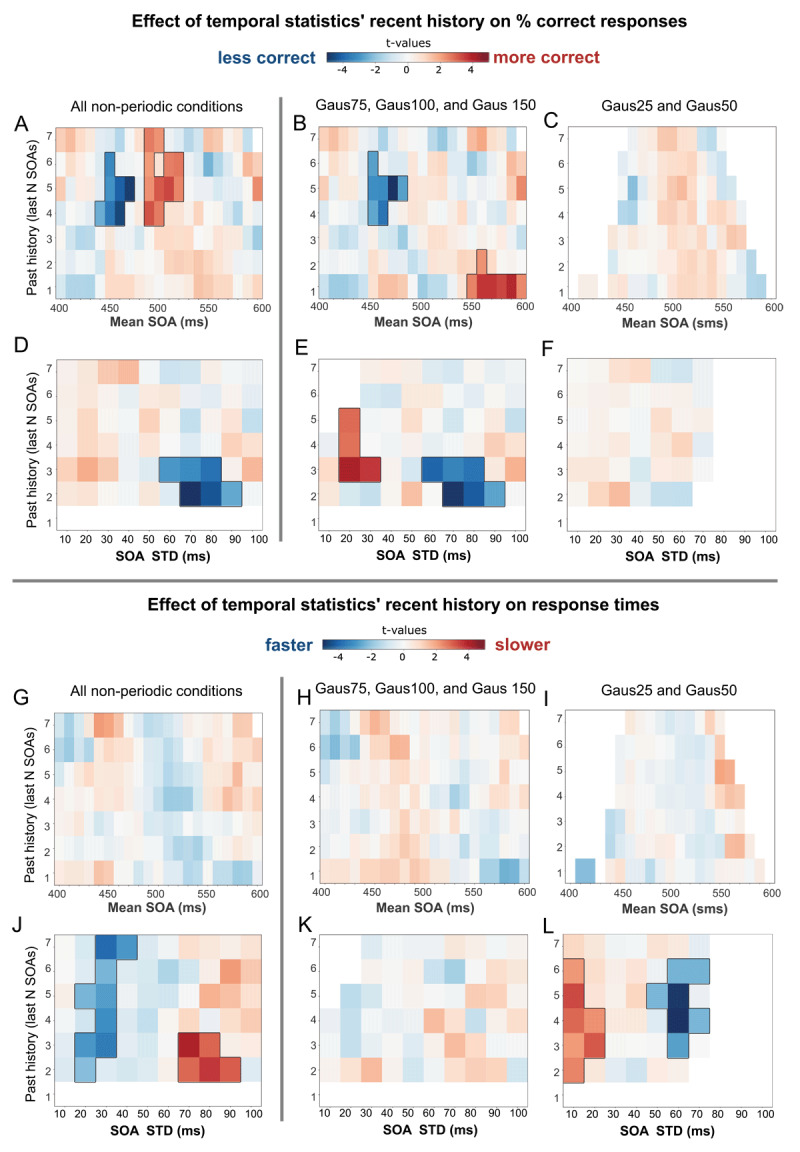
**Effect of local temporal SOAs’ statistics on perception**. The figures illustrate whether the relative performance of participants was affected by the mean and STD of the previous N SOAs. Specifically, we computed the mean and STD of the local SOA distribution drawn from the N previous SOAs before each target trial (with N ranging from 1 to 7 SOAs before target trial for mean SOA, and from 2 to 7 SOAs before target trial for SOA STD). We then binned target trials per SOA distribution mean (from 400 to 600 ms SOA mean, with a sliding window of ±20 ms length) and per SOA distribution STD (from 10 ms to 100 SOA STD, with a sliding window of ±10 ms length). Bins containing less than 5 trials per participant were excluded from further analysis. For each bin, the average accuracy and response time across trials was computed, and then z-scored across participants. The obtained 2-D plots represent whether accuracy and response times were relatively higher or lower depending on the mean and STD of the 2/3/4/… last SOAs. Data were aggregated for either (**A, D, G, J**) all non-periodic contexts, (**B, E, H, K**) the more variable temporal sequences (Gaus75 and higher), and (**C, F, I, L**) the less variable temporal sequences (Gaus25 and Gaus50 conditions). The color label represents the one sample t-test value against zero for each sample. Black lines denote significant clusters (transparency is applied to non-significant areas). Due to the low variability in conditions Gaus25 and Gaus50 some bins are left white (empty) because the number of trials was not sufficient to be representative (<5).

We further investigated the effect of temporal statistics’ recent history in auditory discrimination performances. Specifically, we computed, across all targets in non-periodic sound sequences, the mean and the STD of the distribution of the N-previous SOAs before a target trial (with N ranging from 1 to 7 SOAs for the mean SOA, and N ranging from 2 to 7 SOAs before target trial for the SOA STD), and we asked how the temporal statistics of the N previous SOAs impacted the perception of the target trial. When data in all non-periodic sound sequences were aggregated, auditory discrimination performance was significantly influenced by the mean SOA of N-previous sounds: discrimination accuracy was significantly relatively better when the mean of the last N SOAs was around 500 ms and was significantly worse when the mean of the last N SOAs was around 450 ms (Figure 3A). Moreover, there was a significant effect of preceding local STD on accuracy, performance was relatively worse when the STD of the last SOAs was high (relative decrease in performance most prominently observed for STD around 60–90 ms) (Figure 3D). Response times were not significantly affected by the mean SOA of local context (though the direction of the effect is consistent with the working hypothesis, with a relatively shorter RT when the mean of the last SOAs was around 500 ms) (Figure 3G). Furthermore, when the STD of the last SOAs was low, responses times were significantly faster, and when the local STD was wider response times were significantly slower (Figure 3J).

Data were pooled across all non-periodic sound sequences to maximize the number of trials per bin. Yet, by the way auditory sequences were designed, more target trials in the less temporally variable auditory sequences (e.g. Gauss25 and Gaus50 conditions) had a low STD of the N-previous SOAs, compared to the more variable sound sequences. To limit the effect of global context, we performed the same analyses by dividing the data into two groups: low-temporal variability (Gaus25 and Gaus50) and high-temporal variability (Gaus75, Gaus100, and Gaus150). Grouping the sequences into 2 groups allowed us to have a minimum of trials for each bin of interest. Pooling the data across the more temporally variable conditions (Gaus75, Gaus100 and Gaus150 conditions), performances followed similar observed patterns as across all non-periodic sound sequences: both local SOA mean and STD significantly influenced the discrimination accuracy. Discrimination accuracy was significantly relatively better when the last SOA (past history of N = 1) was longer. Accuracy was significantly lower when the SOA mean of the previous SOAs was lower than the expected mean 500 ms SOA (Figure 3B). Moreover, accuracy was significantly higher when the STD of the previous SOAs was low and was significantly lower for larger STDs (Figure 3E). No significant clusters were found on responses times (Figure 3HK).

For the less variable conditions (Gaus25 and Gaus50 conditions), no significant effects of the mean SOA were observed, though the effects were in the expected direction: accuracy was higher, and RTs were faster when the mean SOA was around the expected 500 ms (Figure 3CI). We observed no conclusive pattern of local STD on accuracy (Figure 3F). However, contrary to our expectations, response times were relatively slower when local temporal variability was low and faster when the local temporal variability was high (Figure 3L). Considering that the analysis compares the relative change in performance per participant as function of local SOA mean and STD, it is possible that the relative change in performance is less important in the low-variable conditions than for the more variable conditions.

**Figure 4:**
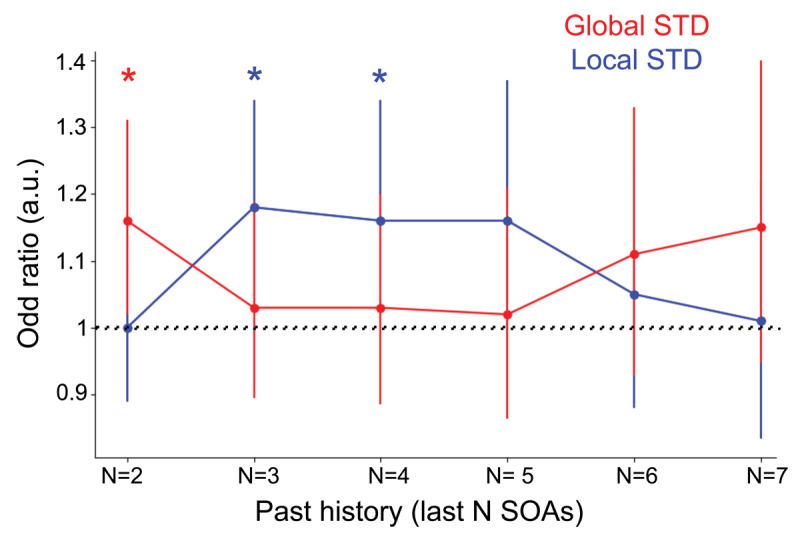
**Interplay between local and global temporal SOAs’ STD on perception**. We ran GLMMs that included both global STD and the local STD (from the N previous SOAs, N rating between 2 to 7) as predictors of subject’s response. The red line denotes the odds ratios of the global STD effect, the blue line denotes the odds ratio of the local STD effect, when local effects are computed with the N previous SOAs, N ranging from 2 to 7. An odds ratio superior to 1 means that participants were more correct for low STD trials than for high STD trials. Bars denote 95% confidence intervals, if the confidence interval is above 1 then the observed STD effects had a significant influence on the subject’s response. The models revealed an interplay between local and global STD effects. The local STD of the N previous SOAs did not have a significant influence on correct responses when N = 2 or N > = 5, while the global STD significantly biased perception. However, for N previous SOAs between 3 and 4 items, we observed that the local STD significantly influenced the subject’s response, while the influence of the global context relatively diminished.

To further evaluate the relative contribution of global and local temporal STD on performances, we ran GLMMs that included both global STD and the local STD (from the N previous SOAs, N rating between 2 to 7), and the last SOA as predictors of subject’s response and response times. The models revealed an interplay between local and global STD effects. The local STD of the N previous SOAs did not have a significant influence on correct responses when N = 2, while the global STD significantly biased perception. However, for N previous SOAs between 3 and 4 items, we observed that the local STD significantly influenced the subject’s response, while the influence of the global context relatively diminished (Figure 4, Supp. Table S1). Similar patterns were observed for response times (Supp. Table S1). This suggests that the local STD could influence performances, and that the observed effect of global STD could partially be explained by the local influence of previous few SOAs.

Altogether, these results suggest that participants integrated the temporal statistics of the global sound sequences. Furthermore, it suggests that the temporal predictions effects at hand were not related to hazard rate. We also investigated whether recent temporal variability affected performances. Both accuracy and response times were affected by local temporal variability: accuracy was significantly improved, and response times were faster when the local temporal variability was low; conversely accuracy was lower and response times are significantly slower when the local temporal context was highly variable. These findings suggest that temporal expectations form quickly, within a few numbers of sounds in the sequence.

### Link between subjective perception of rhythm and auditory discrimination performance

**Figure 5:**
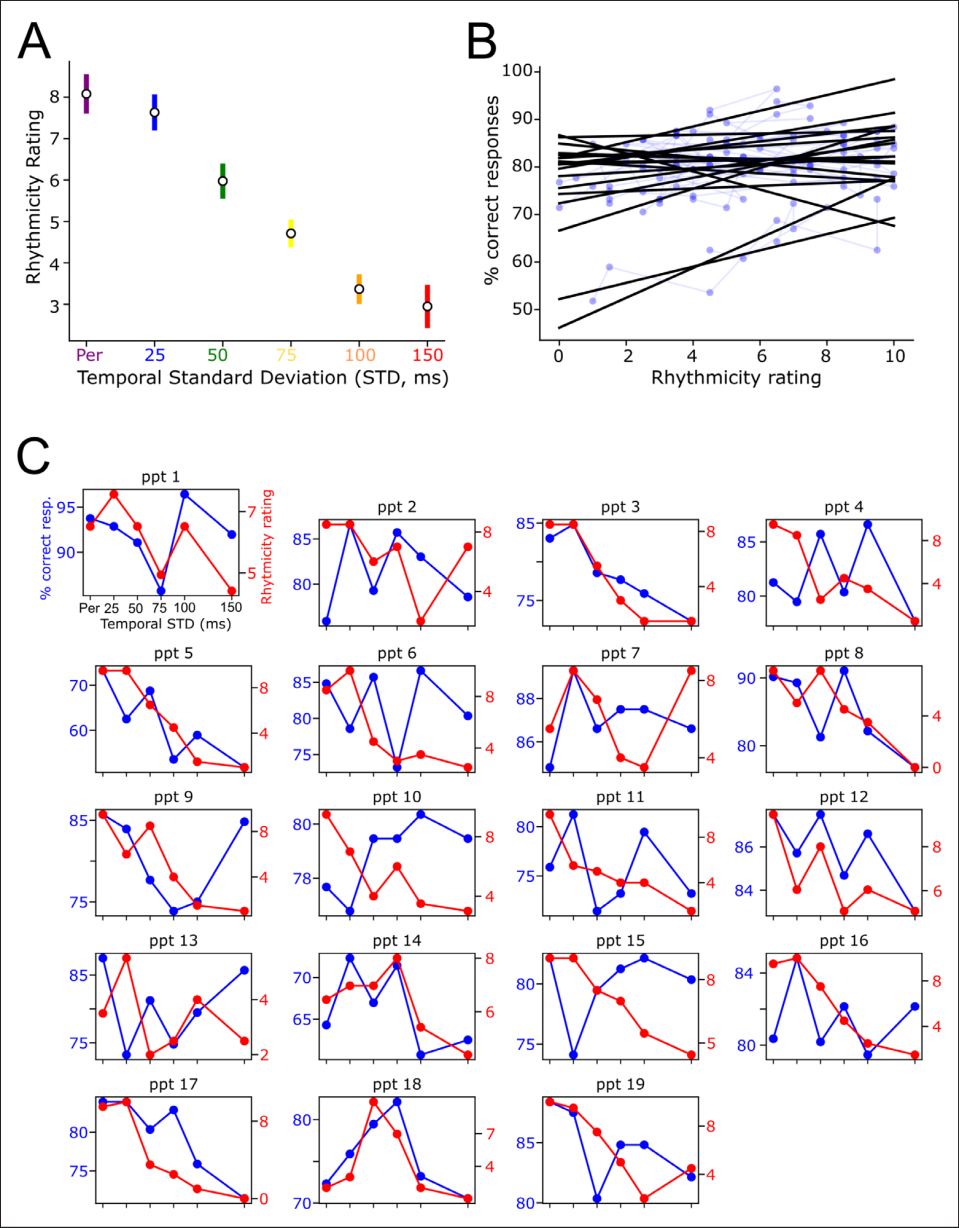
**Link between subjective perception of rhythmicity and auditory performances. (A)** Means of participant’s ratings of the degree of rhythmicity present in the temporal contexts. **(B)** Positive correlation between the rating and the percentage of correct responses. Each point and the corresponding regression line represent a single participant. **(C)** Individual correlations between percentage of correct responses and rhythmicity ratings. Each figure represents a participant’s data. Blue lines denote the percentage of correct response as a function of temporal standard deviation of sound sequences. Red lines denote the subjective rating of rhythmicity of each sound sequence.

We also asked participants to subjectively rate their perception of the rhythmicity of each sound sequence. After each sequence, participants rated from 0 (totally arrhythmic) to 10 (totally periodic) the rhythmicity of the sequence of sounds. Participants rated low-variability sequences as more rhythmic: rhythmicity rating was negatively correlated with the temporal variability of context (main effect of the factor Global STD on Rhythmicity Rating as dependent variable: (*χ2*(1) = 43.364; *p* < 0.0001)) (Figure 5A). Global STD and Subjective rating being highly correlated, Rhythmicity Rating also correlated with the participant’s discrimination accuracy (main effect of Rhythmicity Rating on subjects’ responses: (*χ2*(1) = 14.84; *p* = 0.0001)) (Figure 5B). Yet, inter-subject variability in rating was observed, with some participants rating non-periodic sequences as more rhythmic than periodic sequences (Figure 5C). We therefore investigated whether rhythmicity rating could be a predictor of participants performances, specifically whether adding the Rhythmicity Rating as factor with the model fit would explain away more variance in auditory discrimination performance. However, this was not the case: comparing statistical models with the likelihood ratio test, adding the Rhythmicity Rating as a fixed effect in the model did not significantly improve the data fitting for the percentage of correct responses (*χ2*(1) = 3.24; *p* = 0.072) nor the response times (*χ2*(1) = 0.3054; *p* = 0.624).

## Discussion

The aim of this study was to investigate the impact of temporal prediction mechanisms on auditory perception in probabilistic temporal contexts. For this, participants were asked to discriminate deviant sounds in auditory sequences, whose SOAs between consecutive sounds were drawn from distinct gaussian distributions. All distributions had the same average SOA (500 ms) but different STDs (from 0 ms up to 150 ms). Auditory perception was influenced by the probabilistic temporal regularities of the sound sequences. Deviant discrimination accuracy was highest and response times were fastest when the deviant sounds were presented in periodic sequences as compared to non-periodic sequences, in line with previous findings (Morillon et al., 2016). However crucially, temporal context also influenced auditory discrimination in the non-periodic sound sequences. Deviant discrimination performances slowly decreased when the temporal variability of the auditory sequences increased. Auditory deviant perception was optimal at the average of the SOA distribution of the sequences, suggesting that both influenced by the mean SOA of sequences and by the temporal variability of the last sounds prior to target.

### Probabilistic timing influences auditory perception

This study emphasizes that the temporal variability of the context impacts auditory performance. These findings are in line with the literature that shows that auditory perceptual sensitivity is enhanced when stimuli are presented within periodic streams of sensory events (Rimmele et al., 2011; Henry & Obleser, 2012; Cravo et al., 2013; Ten Oever et al., 2014; Morillon et al., 2016; Ten Oever et al., 2017) or when the temporal context is deterministic (Cope et al., 2012; Morillon et al., 2016; Breska & Deouell, 2017). Our findings further suggest that auditory perception relies on probabilistic inference of events timing. Target discrimination accuracy slowly degraded with increasing temporal variability of sound sequences. Response times showed a plateau effect, where a similar increase in response times duration was observed for the more variable contexts from 75 ms STD as compared to contexts with SOA variability below 75 ms STD. When data were restricted to targets presented at the mean SOA of the distribution, we observed that discrimination performances (both in terms of accuracy and RTs) were relatively better for periodic and for low-temporally variable sound sequences as compared to more temporally variable sequences (of standard deviation above 50 ms/75 ms, i.e. 10-15% of the mean SOA of the distribution). The persistence of contextual effects when restricting analyses to targets presented at the same SOA (500 ms) shows that the effect of temporal context on auditory perception cannot be explained by the sole influence of the last SOA between the target and the preceding sound. In particular, these results cannot be influenced by hazard rate effects (i.e. relatively better performances when targets stimuli occur later than expected, and poorer performances when the target stimuli arrive before than expected (Luce, 1986; Nobre & Van Ede, 2018). Furthermore, these results also rule out the hypothesis that participants only assume a 500-ms SOA, and are surprised when it is not met. If our contextual effects only relied on the assumption of a 500-ms SOA, we would observe similar performances for all targets with a preceding 500 ms SOA. However, we do see that the discrimination performance of these targets decreased when the global temporal context was more temporally variable. We argue that these observations result from probabilistic temporal predictions mechanisms, i.e. that the participant forms an internal model of the distribution of SOAs. Specifically, temporally predictable contexts would give an advantage in target auditory discrimination versus no advantage for no predictable contexts: in a low temporal variability context, participants can leverage temporal predictions to anticipate stimulus arrival, thereby enhancing auditory perception. However, in temporally variable contexts, there is no reliable temporal cues in order to predict the timing of the target sound, therefore temporal predictability cannot benefit auditory discrimination. This suggests that perception is not only influenced by the probabilistic inference of the mean SOA between sounds, but also by the amount of temporal variability of the context.

The effects of probabilistic timing of sensory context have previously been observed on response times (Cannon, 2021; Herbst & Obleser, 2017; Herbst & Obleser, 2019; Grabenhorst et al, 2019; Grabenhorst et al., 2021). In these studies, participants either performed auditory discrimination tasks (Herbst & Obleser, 2017; Herbst & Obleser, 2019), tapping (Cannon, 2021), or “set-go” tasks (Grabenhorst et al., 2019; Grabenhorst et al., 2021; Los et al., 2017). The temporal distribution of the foreperiod before the target stimuli influenced response times in both tasks. Importantly, like in this current study, temporal expectancy mechanisms were not uniquely driven by the hazard rate of events, but were also sensitive to the probability of events timing so that response times were fastest when events occur around the mean of contextual temporal probability distribution. Our results further show that not only response times, but also auditory perceptual sensibility is influenced by the temporal probabilities of contextual information. They also highlight that the time required to implement temporal predictions mechanisms based on contextual temporal probabilities is relatively short (temporal statistics from the previous 3 SOAs can already bias target perception and response times) and depends on the degree of confidence in the temporal regularities of the context.

Knowing that the perception of rhythmic cues in auditory signals is variable across individuals (Potter et al., 2009, Obleser et al., 2017) and could depend on several factors, such as the participant’s musical expertise (Geiser et al., 2009), we examined whether participants accurately perceived the amount of temporal variability in the sound sequences, and whether this impacted their performances. Participants accurately assessed the amount of temporal regularity in the sound sequences, as their subjective rating of temporal regularity slowly decreased with increased temporal STD of the context. Interestingly, individual variability in the ratings were observed, so that certain participants rated more variable temporal contexts as more rhythmic. However, subjective variations in the perception of rhythmic cues did not significantly add explanatory power to the auditory performances.

### Putative neural mechanisms behind probabilistic temporal predictions

The present findings have implications for current theories and frameworks linking low-frequency neural oscillations to temporal prediction mechanisms in auditory perception. The neural entrainment theory postulates that external rhythms can entrain endogenous neural oscillations, which reflect periodic fluctuations in excitability of neuronal populations (Schroeder & Lakatos, 2009; Cravo et al., 2013). According to this view, neural excitability fluctuations temporally align to the periodic external stream so that the period of high neural excitability coincides with the beat of the external stimuli. A direct prediction of the neural entrainment theory is that perception should be optimal when stimuli are periodic enough to entrain neural oscillations, and when target sensory events occur on beat with the entrained neural oscillation. Here, we do actually report that temporal prediction mechanisms do not only account for purely periodic stimuli but are also robust to a certain amount of temporal variability. Specifically, we found that temporal predictions benefit auditory perception until the variability of temporal context reaches a STD threshold of 10–15% of the mean SOA of the distribution. It is possible that the neural entrainment theory, tested in periodic contexts, could generalize to more complex temporal predictions observed in hierarchically structured rhythms (e.g., speech or music). Importantly, this would explain why temporal properties of speech signals, which are not periodic (Nolan & Jeon, 2014) but are still based on probabilities of occurrence, influence the perceived duration of speech segments and neural dynamics in auditory cortices (Kösem et al., 2018). Interestingly, a recent computational model reports that neural oscillators can handle a certain degree of temporal variability: Stuart–Landau neural oscillatory models are still able to synchronize to temporally variable stimuli, with SOAs drawn from Gaussian distributions with standard deviations going up to 20% of the mean SOA (Doelling & Assaneo, 2021). Empirically, neural entrainment is observable for stimuli that are not fully isochronous (Calderone, 2014; Lakatos et al., 2008; Herrmann et al., 2016; Kösem et al., 2018). Yet, it is still unclear what exact degree of temporal variability entrainment mechanisms can handle. Interestingly, local variations of sounds sequences’ timing can affect neural entrainment to the delta range (Herrmann et al., 2016): entrained delta oscillations to a temporally variable sound sequences were shown to fluctuate in amplitude over the course of the sequence. Importantly, the phase of entrained delta oscillations was indicative of sound deviant discrimination, but only when the delta entrainment was strong. It is possible that, in this experiment, epochs with high delta oscillatory activity were corresponding to periods where the sound sequences were sufficiently temporal regular and predictable so that entrainment could occur, while epochs with low delta activity were reflecting failure of neural oscillatory activity to entrain to more temporally variable contexts (Herrmann et al., 2016).

Alternatively, the temporal predictions mechanisms observed in periodic and probabilistic contexts could rely on low-frequency dynamics, but would not obviously reflect neural entrainment per se. Evidence for this hypothesis is that low-frequency neural dynamics are shown to reflect temporal predictions in non-entrained sensory context, e.g. when temporal predictions rely on memory-based patterns (Wilsch et al., 2015; Breska & Deouell, 2017; Daume et al., 2021; Herbst et al., 2022). However, it is possible that memory-based predictions and temporal contextual predictions may rely on different co-existing neural mechanisms (Bouwer et al., 2020; Bouwer et al., 2022). The results of this study also support the view that predictive probabilistic timing is more than hazard rate, and that it also relies on the probability density function of the timing of previous sensory events. As such, the mechanisms related to hazard rate and contextual temporal predictions could be dissociated and have different mechanistic origins. While hazard rate processing seems to rely on motor regions (Herbst et al., 2018; Cui et al., 2009), contextual probabilistic timing may involve a different distributed neural architecture, including early sensory areas (Bueti et al., 2010; Herbst et al., 2018).

In our study, we presented visual cues in synchrony with the auditory sounds. The visual cue was indicating the target sound, so as to make sure that the response of the participant was referring to the target, and not to any other sound from the sequence. A limitation from this manipulation is that visual timing could potentially have affected the temporal precision of the observed auditory effects, considering that the visual event timing can interact with the perception of auditory event timing (Di Luca & Rhodes, 2016), and that event timing of visual events are usually judged with less precision than auditory events (Di Luca & Rhodes, 2016; Zalta et al., 2020), though audition is known to dominate the temporal judgments of audiovisual stimuli (Wilsch et al. 2020), e.g. during rhythmic stimulation where perceived audiovisual rates are usually shifted towards the auditory rate (Wada et al., 2003; Welch et al., 1986). Visual timing also interacts with neural entrainment mechanisms, so that visual stimuli can modulate the phase of entrainment of auditory cortices (Lakatos et al., 2008; Kösem et al., 2014). We cannot fully conclude whether the observed temporal predictions mechanisms rely on unimodal or crossmodal mechanisms. However, the present results provide evidence that temporal predictive mechanisms influence auditory perception in implicit probabilistic temporal contexts, and that they are robust to some amount temporal variability.

## Data Accessibility Statements

Data and code are available for peer-review at the following link: https://researchbox.org/1539&PEER_REVIEW_passcode=TWKWIZ.

## Additional Files

The additional files for this article can be found as follows:

10.5334/joc.344.s1 Table s1. Models summaries of local statistics vs global statistics influences on subject’s responses and subject’s response times.

10.5334/joc.344.s2 Figure s1. Data exclusion criteria.
