# Supplementary material for: What is a Rhythm for the Brain? The Impact of Contextual Temporal Variability on Auditory Perception
Source: J Cogn. 2024 Jan 17;7(1):15. doi: 10.5334/joc.344 (PMC10798173; doi:10.5334/joc.344)
Supplement: Table s1. — Models summaries of local statistics vs global statistics influences on subject’s responses and subject’s response times. [file joc-7-1-344-s1.pdf]

**Model: Dependent variable ~GlobalSTD + LastSOA + LocalSTD + (1+ GlobalSTD ∣ Subject)**								
Local STD from N previous SOAs								
**N = 2**								
	**Dep. Var.: subject's responses**				**Dep. Var.: response times**			
**Groups**	Name	Variance	SD	Corr	Name	Variance	SD	Corr
**Subj**	(Intercept)	0.1285	0.3585		(Intercept)	0.00183	0.04278	
	global STD	1.2459	1.1162	0.48	Global STD	0.04278	0.21018	-0.39
**Residual**						0.09853	0.31390	
**Fixed effects:**								
	**Estimate**	**SE**	**Z value**	**Pr(>|z|)**	**Estimate**	**SE**	**t value**	**Pr(>|t|)**
**Intercept**	1.15013	0.15962	7.205	**< 0 .001**	0.61127	0.02786	21.938	**< 0.001**
**Global STD**	-1.46183	0.63695	-2.295	**0.02173**	0.19790	0.09112	2.172	**0.0299**
**Last SOA**	0.75670	0.25905	2.921	**0.00349**	-0.03023	0.01765	-1.713	**0.0868**
**Local STD**	-0.02454	0.60973	-0.040	0.96790	-0.02721	0.04182	-0.651	0.5153
**N = 3**								
**Groups**	Name	Variance	SD	Corr	Name	Variance	SD	Corr
**Subj**	(Intercept)	0.1285	0.3584		(Intercept)	0.001828	0.04276	
	Global STD	1.2599	1.1224	0.48	Global STD	0.044112	0.21003	-0.39
**Residual**						0.098496	0.31384	
**Fixed effects:**								
	**Estimate**	**SE**	**Z value**	**Pr(>|z|)**	**Estimate**	**SE**	**t value**	**Pr(>|t|)**
**Intercept**	1.1712	0.1582	7.401	**< 0 .001**	0.61008	0.02789	21.876	**< 0.001**
**Global STD**	-0.3017	0.7222	-0.418	0.67615	0.10619	0.09370	1.133	0.2571
**Last SOA**	0.7147	0.2556	2.796	**0.00518**	-0.02779	0.01783	-1.558	0.1192
**Local_STD**	-1.6233	0.6676	-2.431	**0.01504**	0.10590	0.04669	2.268	**0.0233**
**N = 4**								
**Groups**	Name	Variance	SD	Corr	Name	Variance	SD	Corr
**Subj**	(Intercept)	0.1285	0.3585		(Intercept)	0.001829	0.04277	
	Global STD	1.2569	1.1211	0.48	Global STD	0.044133	0.21008	-0.39
**Residual**						0.098534	0.31390	
**Fixed effects:**								
	**Estimate**	**SE**	**Z value**	**Pr(>|z|)**	**Estimate**	**SE**	**t value**	**Pr(>|t|)**
**Intercept**	1.1635	0.1584	7.347	**< 0 .001**	0.61070	0.02787	21.909	**< 0.001**
**Global STD**	-0.3167	0.7827	-0.405	0.68574	0.14078	0.09642	1.460	0.144
**Last SOA**	0.7336	0.2559	2.867	**0.00414**	-0.02911	0.01773	-1.642	0.101
**Local STD**	-1.4981	0.7434	-2.015	**0.04387**	0.05331	0.05210	1.023	0.306

**N = 5**								
**Groups**	Name	Variance	SD	Corr	Name	Variance	SD	Corr
**Subj**	(Intercept)	0.1285	0.3585		(Intercept)	0.001829	0.04276	
	Global STD	1.2559	1.1206	0.48	Global STD	0.044066	0.20992	-0.39
**Residual**						0.098528	0.31389	
**Fixed effects:**								
	**Estimate**	**SE**	**Z value**	**Pr(>|z|)**	**Estimate**	**SE**	**t value**	**Pr(>|t|)**
**Intercept**	1.1714	0.1587	7.380	**< 0 .001**	0.60997	0.02788	21.876	**< 0.001**
**Global STD**	-0.2257	0.8598	-0.262	0.79294	0.10573	0.09941	1.064	0.288
**Last SOA**	0.7175	0.2567	2.795	**0.00519**	-0.02765	0.01780	-1.553	0.120
**Local STD**	-1.5221	0.8197	-1.857	0.06333	0.09259	0.05737	1.614	0.107

**N = 6**								
**Groups**	Name	Variance	SD	Corr	Name	Variance	SD	Corr
**Subj**	(Intercept)	0.1285	0.3585		(Intercept)	0.001829	0.04276	
	Global STD	1.2478	1.1171	0.48	Global STD	0.044017	0.20980	-0.39
**Residual**						0.098540	0.31391	
**Fixed effects:**								
	**Estimate**	**SE**	**Z value**	**Pr(>|z|)**	**Estimate**	**SE**	**t value**	**Pr(>|t|)**
**Intercept**	1.1553	0.1592	7.255	**< 0 .001**	0.61020	0.02788	21.885	**< 0.001**
**Global STD**	-1.0614	0.9234	-1.149	0.25039	0.09016	0.10255	0.879	0.3793
**Last SOA**	0.7475	0.2579	2.898	**0.00375**	-0.02811	0.01780	-1.580	0.1142
**Local STD**	-0.4897	0.8996	-0.544	0.58616	0.10752	0.06287	1.710	0.0872

**N = 7**								
**Groups**	Name	Variance	SD	Corr	Name	Variance	SD	Corr
**Subj**	(Intercept)	0.1285	0.3585		(Intercept)	0.001829	0.04277	
	Temporal STD	1.2460	1.1162	0.48	Temporal STD	0.044062	0.20991	-0.39
**Residual**						0.098546	0.31392	
**Fixed effects:**								
	**Estimate**	**SE**	**Z value**	**Pr(>|z|)**	**Estimate**	**SE**	**t value**	**Pr(>|t|)**
**Intercept**	1.1505	0.1595	7.214	**< 0 .001**	0.61024	0.02788	21.888	**< 0.001**
**Global STD**	-1.4124	0.9912	-1.425	0.15418	0.09790	0.10643	0.920	0.358
**Last SOA**	0.7561	0.2586	2.924	**0.00345**	-0.02807	0.01778	-1.579	0.114
**Local STD**	-0.0722	0.9585	-0.075	0.93996	0.09454	0.06823	1.386	0.166

SD; standard deviation; SE, standarderror; Corr, correlation

Table S1: Models summaries of local statistics vs global statistics influences on subject's responses and subject's response times
