# Supplementary material for: What is a Rhythm for the Brain? The Impact of Contextual Temporal Variability on Auditory Perception
Source: J Cogn. 2024 Jan 17;7(1):15. doi: 10.5334/joc.344 (PMC10798173; doi:10.5334/joc.344)
Supplement: Figure s1. — Data exclusion criteria. [file joc-7-1-344-s2.pdf]

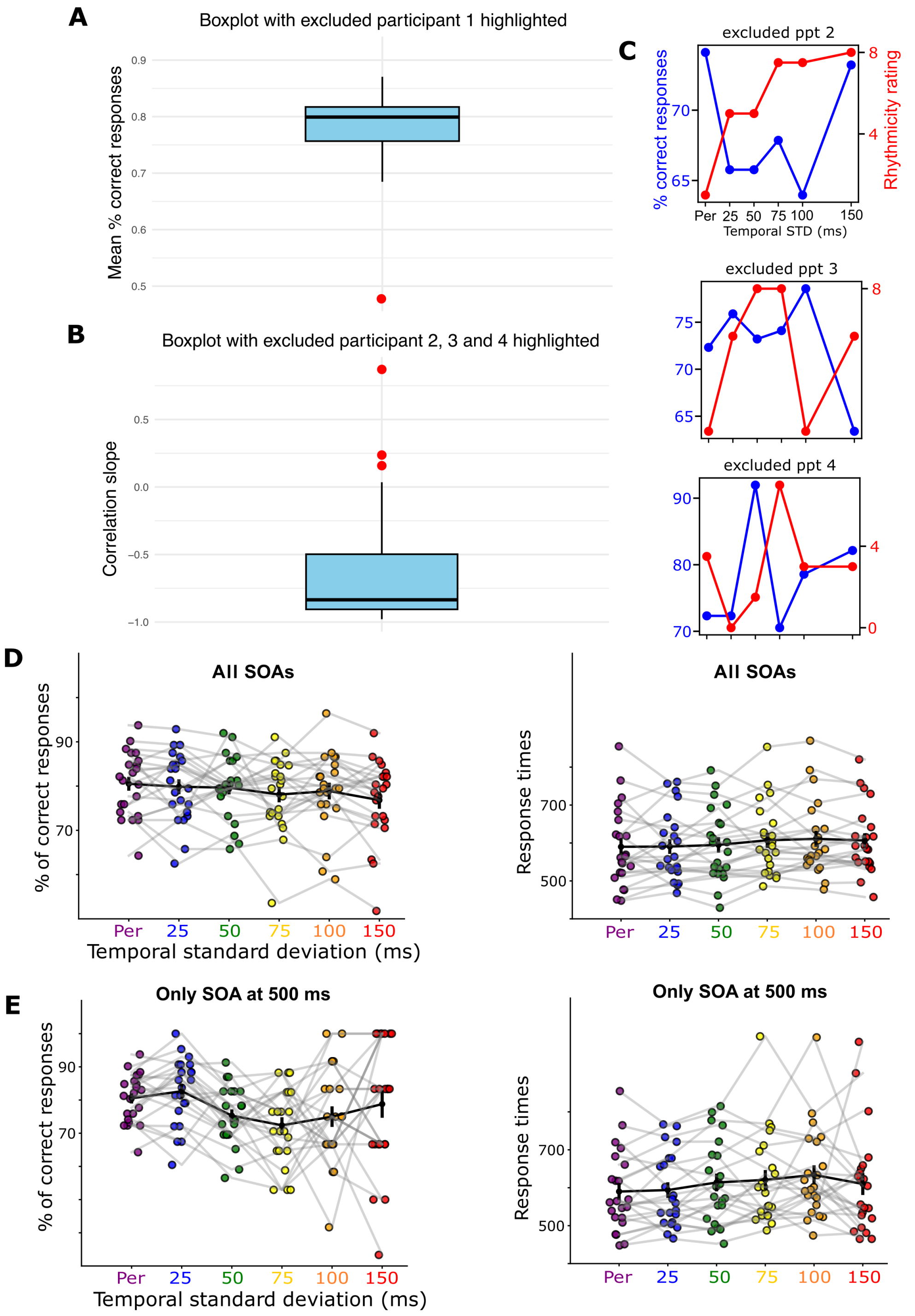

Figure S1: Data exclusion criteria. Four participants had outlier data and were excluded from data analysis. (A) one participant responded at chance level throughout the experiment (highlighted in red) (B) three participants had outlier subjective rhythmicity ratings of the sequences (±1.5 interquartile range of regression scores, highlighted in red). (C) Response and rhythmicity rating of the three excluded participants from (B). (D-E) Including the three participants from (B) does not influence the main patterns of results. (D) We still observe a main effect of the global Temporal STD on the correct responses ($\chi2(1) = 12.17$, $p = 0.00048$) and on response times ($\chi2(1) = 17.909$, $p < 0.0001$). (E) When we restrict the analysis to targets presented at 500 ms SOAs, we also have a main effect of global Temporal STD on the correct responses ($\chi2(1) = 14.49$, $p = 0.00014$) and on response times ($\chi2(1) = 26.468$, $p < 0.0001$).
